# Physicochemical Characterization of Thirteen Quinoa (*Chenopodium quinoa* Willd.) Varieties Grown in North-West Europe—Part II

**DOI:** 10.3390/plants11030265

**Published:** 2022-01-19

**Authors:** Phara De Bock, Gerda Cnops, Hilde Muylle, Paul Quataert, Mia Eeckhout, Filip Van Bockstaele

**Affiliations:** 1 Research Unit of Cereal and Feed Technology, Department of Food Technology, Safety and Health, Faculty of Bioscience Engineering, Ghent University, 9000 Ghent, Belgium; mia.eeckhout@ugent.be; 2 Plant Sciences Unit, Flanders Research Institute for Agriculture, Fisheries and Food (ILVO), 9090 Melle, Belgium; gerda.cnops@ilvo.vlaanderen.be (G.C.); hilde.muylle@ilvo.vlaanderen.be (H.M.); paul.quataert@ilvo.vlaanderen.be (P.Q.); 3 Food Structure and Function Research Group (FSF), Department of Food Technology, Safety and Health, Faculty of Bioscience Engineering, Ghent University, 9000 Ghent, Belgium; filip.vanbockstaele@ugent.be

**Keywords:** *Chenopodium quinoa* Willd., North-West Europe, wholemeal flour, water holding capacity, water absorption index, pasting behavior

## Abstract

Quinoa cultivation has gained increasing interest in Europe but more research on the characteristics of European varieties is required to help determine their end use applications. A comparative study was performed on 13 quinoa varieties cultivated under North-West European field conditions during three consecutive growing seasons (2017–2019). The seeds were milled to wholemeal flour (WMF) to evaluate the physicochemical properties. The WMFs of 2019 were characterized by the highest water absorption capacity (1.46–2.06 g/g), while the water absorption index (WAI) between 55 °C (2.04–3.80 g/g) and 85 °C (4.04–7.82 g/g) increased over the years. The WMFs of 2018 had the highest WAI at 95 °C (6.48–9.48 g/g). The pasting profiles were characterized by a high viscosity peak (1696–2560 mPa.s) and strong breakdown (−78–643 mPa.s) in 2017. The peak viscosity decreased in 2018 and 2019 (823–2492 mPa.s), while breakdown (−364–555 mPa.s) and setback (19–1037 mPa.s) increased. Jessie, Summer Red, Rouge Marie, Vikinga, and Zwarte WMFs were characterized by low WAIs and high shear resistance. Bastille WMF developed high viscosities and, along with Faro WMF, showed a high breakdown. The wide variation in physicochemical properties suggests that the potential food applications of WMFs depend on the variety and growing conditions.

## 1. Introduction

The pseudocereal quinoa (*Chenopodium quinoa* Willd.) belongs to the Amaranthaceae family. Quinoa was traditionally cultivated by pre-Hispanic civilizations in the Andean region [1]. The plant produces edible seeds that are small, flat, and round [2]. The seed color of most commercial varieties are white to grey or black but varieties with yellow or red–violet seeds exist as well [3,4]. After the recognition of its excellent nutritional value, quinoa gained increasing interest worldwide [1]. Quinoa is a promising crop for further expansion in many parts of the world [2]. Quinoa is mainly grown in South America (e.g., Peru, Bolivia, Ecuador), although it has been introduced worldwide in the last decades [5].

Quinoa is known for its excellent balance between carbohydrates, proteins, and lipids [6,7]. Quinoa’s carbohydrate fraction mainly consists of starch (32–69 g/100 g), with a significant amount of resistant starch and dietary fiber (7.0–11.7 g/100 g) [5,6]. Albumins and globulins make up the main fraction of the total protein content (7–23 g/100 g). Quinoa is gluten-free, making it a suitable food source for people with celiac disease or other digestive disorders, such as gluten intolerance [6]. Quinoa proteins have a balanced composition of essential amino acids [7]. The protein fraction shows a high content of lysine, methionine, and threonine, the limiting amino acids in common cereals, such as wheat and maize [5]. All essential amino acids are present, meeting the requirements for adults suggested by FAO and WHO [5]. The protein digestibility is usually high, but may be affected by hydrolase inhibitors or enzyme inhibitory effects of endogenous phenolic compounds [6]. Besides its nutritional interesting protein content, quinoa has an interesting lipid content of approximately 5.5 to 7.4 g/100 g, which is higher than that of wheat, maize, and rice [5]. Quinoa seeds have a higher content of potassium, calcium, magnesium, iron, copper, and zinc than those found in common cereals [5]. Quinoa is a good source of vitamins with higher levels of α-tocopherol (vitamin E), riboflavin (B2), pyridoxine (B6), and folic acid than those of wheat [5]. Quinoa contains several antioxidants and phytochemicals with positive effects on human health and nutrition [5,8].

As starch is the major component in quinoa seed, it is expected that the properties of quinoa wholemeal flour (WMF) depend, to a large extent, on the composition and properties of starch [9]. Quinoa starch is present in the form of small polygonal granules of 1 to 3 µm in diameter [7,9,10]. The starch granules are present as single units or as spherical aggregates packed in the quinoa perisperm [7,11]. Quinoa starch has a low amylose content, which provides different physicochemical properties compared to cereal starches [12]. There is a considerable variability in the amylose content of quinoa [13]. The amylose content reported for quinoa starch ranges between 9 and 11%, although wider ranges of 3 to 20% have also been found [12,13]. It is unclear whether the reported variation in amylose content is truly a reflection of genetic variability, due to variations in agricultural practices or environment, the result of both, or attributable to differences in the quantification methods [13]. Quinoa amylopectin contains a large number of short chains from 8 to 12 units and a small number of longer chains from 13 to 20 units, as compared to the endosperm starches of cereals [13,14]. The low amylose content and unique amylopectin structure contribute to the unique physicochemical characteristics of quinoa starch [2,11]. It gelatinizes at relatively low temperatures, similar to the gelatinization temperature of wheat and potato starch. However, its pasting behavior is considerably different from that of wheat and potato starches [7,13]. Quinoa starch shows a higher water-binding capacity and swelling power compared to wheat, barley, or corn starch. Furthermore, it has excellent freeze–thaw stability [7,11,12,13]. Other main seed components, such as proteins and lipids, likely affect the physicochemical properties of the quinoa WMF as well [9]. Proteins can affect the water-binding capacity, pasting properties, and digestibility of the WMF [9]. Polar lipids could form V-type inclusion complexes with amylose, which influences starch gelatinization, retrogradation, and susceptibility to enzymatic breakdown [9]. Non-starch polysaccharides, such as dietary fiber, could increase gel viscosity or form a matrix with phenolic compounds, which may retard starch retrogradation and digestion [9].

The wide applicability of quinoa derives from its versatility as a food ingredient [1]. Quinoa is mostly consumed as a whole grain or milled as WMF [1]. As a whole grain, quinoa seeds are toasted, puffed, or boiled, and used in salads, cooked meals, breakfast, or soups [1,15]. The seeds can also be processed into dairy milk, fermented to make beer, or used as an ingredient in the traditional chicha drink [15,16]. Quinoa WMF, whether or not combined with wheat flour or corn meal, is mainly used for the production of baked (biscuits, cookies, bread, and gluten-free bakery products) or cooked (pasta, noodles) products [2,17]. However, end-product quality will greatly depend on the characteristics of the flour used [1]. According to previous studies, different varieties of quinoa have shown different results in similar food products [2]. Therefore, studying the physicochemical properties is important in determining the end uses of quinoa varieties, as these properties determine the changes that occur during processing [2].

The significant increase in demand for quinoa has stimulated efforts in Europe to develop new varieties that are suited to European growing conditions [2,18]. The characteristics of Andean quinoa varieties are relatively well studied, but far little information is available about European quinoa [1]. A better understanding of the individual variety characteristics will facilitate the selection of a variety for specific markets or products and will stimulate the utilization of European quinoa in the food industry [2]. To fill the gap in the current literature on European varieties and to assess their potential usefulness for farmers and end users, a comparative variety testing with ten European bred varieties and three Farm Original varieties (USA) was performed under field conditions in North-West Europe during three consecutive growing seasons (2017–2019). In a previous work [19], the agronomic performances of these varieties were evaluated, and the quinoa seeds were qualitatively characterized based on physical and nutritional properties. The present work focuses on the characterization of the physicochemical properties of WMFs obtained from these seeds. These properties were evaluated over the three growing seasons to estimate the impact of varying seed composition. In addition, principal components analysis (PCA) and hierarchical cluster analysis (HCA) were applied to group the WMFs into clusters with similar physicochemical properties.

## 2. Results and Discussion

### 2.1. Chemical Composition

The chemical composition of the quinoa varieties is summarized in Table 1 and visualized in Supplementary Materials (Figure S1). These data have been discussed in detail in Part I of this study [19]. In brief, the protein content of the quinoa seeds varied between 12.1 and 18.8 g/100 g dry matter (dm). All varieties obtained the highest protein concentration in 2019 due to favorable weather conditions. Among varieties, Atlas and Pasto were characterized by the highest protein content in combination with lower yields. Quinoa seeds contained a considerable amount of fat (5.42–8.54 g/100 g dm), with the highest levels measured in 2018. The seeds of Pasto, Puno, and Zwarte usually had lower fat levels than other varieties, while the highest amounts of fat were found in Rouge Marie, Summer Red, and Vikinga seeds. The main component of the quinoa seeds was starch (50.5 to 72.5 g/100 g dm). Jessie, Pasto, Titicaca, Vikinga and Zwarte obtained the lowest starch content in 2017, other varieties in 2019. Among varieties, Puno and Titicaca contained the highest amounts of starch, Faro the lowest. The quinoa seeds contained between 2.37 and 3.60 g minerals per 100 g dm. The seeds of 2019 usually had a lower ash content compared to corresponding seeds from 2017 or 2018. Puno and Titicaca contained low amounts of minerals, while Pasto seeds were characterized by the highest ash content.

### 2.2. Physicochemical Properties

#### 2.2.1. Water Absorption Capacity

**Figure 1:**
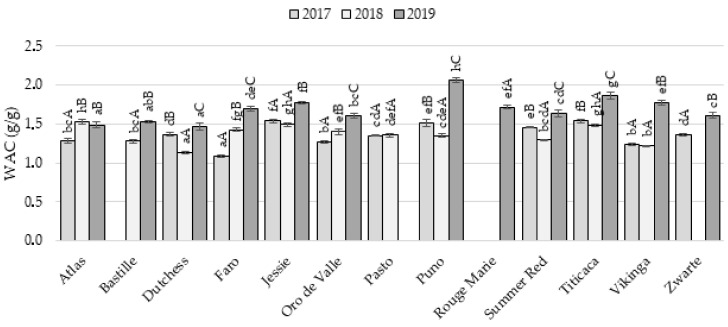
Water absorption capacity (WAC, g/g) of 13 quinoa wholemeal flours obtained from seeds cultivated under North-West European field conditions in 2017, 2018 and 2019 (*n* = 3). Within years, average values followed by the same lowercase letter are not significantly different (*p* > 0.05). Capital letters compare the three years for the same variety, average values followed by the same letter are not significantly different (*p* > 0.05).

The water absorption capacity (WAC) is the ability of the WMF to physically hold water while exposed to a centrifugal force, a process in which macromolecules (e.g., carbohydrates and proteins) increase the water absorption by offering hydrophilic side chains [17,20]. Overall, the WAC ranged between 1.08 and 2.06 g/g (Figure 1), which corresponded with the findings of Ghumman et al. (1.22–2.17 g/g, [17]) and De Bock et al. (1.52–2.05 g/g, [20]). Vázquez-Luna et al. [21] reported a higher WAC for quinoa flour (2.11–2.55 g/g), while the WAC was lower according to Aluwi et al. (0.89–1.22 g/g, [2]). The growing season of the quinoa seeds had a significant impact on the WAC of the WMFs. The WAC of Faro, Oro de Valle, and Zwarte WMF improved every growing season, while other WMFs, except for Atlas, Jessie, Pasto, and Vikinga, obtained the lowest WAC in 2018. Jessie, Pasto, and Vikinga WMF showed a stable WAC during the first two seasons (i.e., 2017 and 2018). The WMFs of 2019 showed the highest ability to absorb and retain water, as the WAC varied between 1.46 and 2.06 g/g. The high protein levels in 2019 positively affected the WAC of the WMFs (*r* = 0.563, *p* = 0.001), as high protein contents lead to an improved potential in binding capacity [22,23]. However, the maximum WACs in 2019 were not linked to the WMFs with the highest protein content. Despite the high protein content, Atlas and Dutchess WMF were characterized by the lowest WAC in 2019. Vikinga, Jessie, Titicaca, and Puno, all WMFs rich in starch and low in minerals, showed the highest WAC for WMFs in 2019. The WAC might also be affected by the protein composition or the presence of fiber [23]. Moreover, differences in particle size and damaged starch content could also have an impact on the WAC [1,24]. Differences in WAC between varieties varied among growing seasons (variety × year: *p* < 0.001, Table S4). For example, Atlas was considered a WMF with low WAC in 2017 and 2019 whereas it had the highest WAC among WMFs in 2018. However, certain trends were observed over the different years. Jessie and Titicaca WMFs were generally characterized by a high WAC, while Bastille WMF showed a low capacity to absorb and retain water. WMFs from Pasto or dark colored seeds (i.e., Rouge Marie, Summer Red, and Zwarte) could be considered WMFs with medium–high WAC.

#### 2.2.2. Water Absorption Index

**Figure 2:**
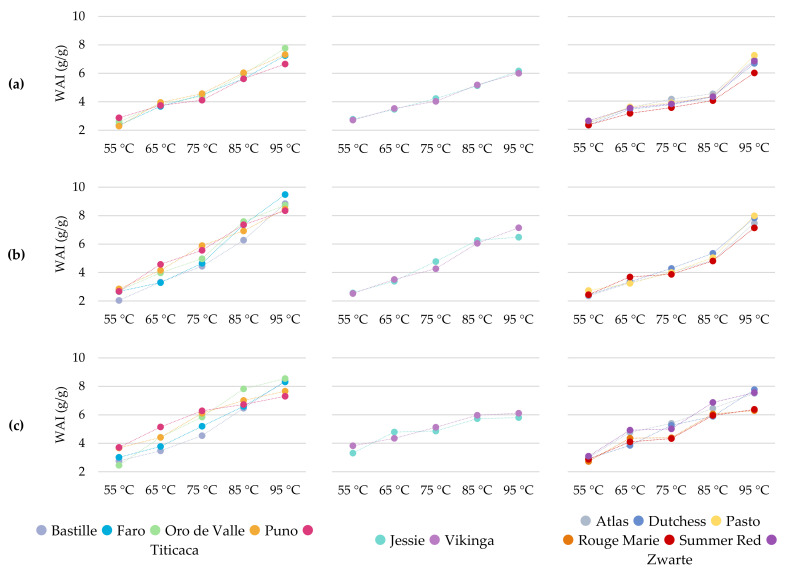
Water absorption index at 55, 65, 75, 85, and 95 °C (WAI, g/g) of 13 quinoa wholemeal flours obtained from seeds grown under North-West European field conditions in 2017 (**a**), 2018 (**b**), and 2019 (**c**) (*n* = 3).

The water absorption index (WAI) measures a WMF’s ability to absorb water and swell, and is a function of the gel-forming capacity of the WMF. It is in fact a property related to the characteristics of a thickening agent, as a high WAI characterizes a good binder [2,23]. The WAI was evaluated over a temperature range between 55 and 95 °C (Figure 2) and was significantly affected by a variety × year interaction (*p* < 0.001, Table S4). The increase in temperature had a positive impact on the WAI of the WMFs, as it promoted starch gelatinization and protein denaturation [1,23,25]. A steady increase in swelling behavior with rising temperature has been observed before [9,20]. Furthermore, more differentiation between the WMFs was observed at higher temperatures. 

The WMFs of 2017 were arranged into three groups based on their swelling behavior (Figure 2). The first group consisted of WMFs with a high ability to swell, namely Faro, Oro de Valle, Puno, and Titicaca. At 65 °C, these WMFs already showed the strongest swelling behavior among all WMFs. The WAI only slightly increased at 75 °C but was still higher for Faro, Puno, and Oro de Valle compared to that of other WMFs. The largest increase in WAI was observed between 75 and 95 °C, where Titicaca WMF usually had the lowest WAI among these four WMFs. The WAI at 95 °C ranged between 7.25 and 7.76 g/g for Faro, Oro de Valle, and Puno (Table S1) and was higher compared to the WAI of other WMFs, except for Pasto WMF. Jessie and Vikinga WMF formed a second group among the WMFs of 2017. These WMFs had a higher WAI at 55 °C than most other WMFs. The WAI of Jessie and Vikinga WMF showed a continuous increase with temperature, eventually reaching the lowest WAI at 95 °C among all samples. The remaining WMFs, i.e., Atlas, Dutchess, Pasto, Summer Red, and Zwarte, were characterized by the lowest WAI at 85 °C, as the WAI increased only slightly between 65 and 85 °C. The strong improvement at 95 °C eventually led to a medium-high WAI for Atlas, Dutchess, and Zwarte WMF (6.68–6.94 g/g) and a high WAI for Pasto WMF (7.25 g/g, Table S1). Summer Red WMF showed a low WAI over the whole temperature range, but its WAI also showed a strong increase at 95 °C (6.00 g/g, Table S1).

Similar observations were found for the 2018 samples, although the first group was separated into two subgroups (Figure 2). The WAI of Puno and Titicaca WMF showed a continuous increase over the whole temperature range. At temperatures above 55 °C, the WAI of these WMFs was higher compared to that of the corresponding WMFs of 2017. Puno and Titicaca WMF were characterized by the highest WAI at 65 and 75 °C and also showed a high swelling behavior at higher temperatures. The WAI of Bastille, Faro, and Oro de Valle increased at a higher rate between 75 and 95 °C, when compared to the increase between 55 and 75 °C. The swelling behavior of Faro and Oro de Valle WMF was generally higher compared to that of the corresponding WMFs of 2017. However, Faro and Oro de Valle were considered WMFs with a high swelling behavior between 65 and 95 °C in 2017. In 2018, the WAI of Faro and Oro de Valle was only high compared to that of other WMFs at 85 and 95 °C. Between 55 and 65 °C, Jessie and Vikinga WMF showed a similar swelling behavior, but the WAI of Jessie was higher at 75 and 85 °C. However, the WAI of Jessie WMF deflected at 95 °C, resulting in a lower WAI than Vikinga. Nevertheless, both WMFs showed a low swelling behavior at 95 °C (6.48–7.14 g/g, Table S1) compared to most other WMFs (7.47–9.48 g/g, Table S1). Moreover, other studies reported a higher WAI at 95 °C for quinoa flour (8.11–9.75 g/g, [19]; 7.89–9.46 g/g, [9]). The swelling behavior of Jessie and Vikinga followed the same trend as observed in 2017, although the WAIs at 75 to 95 °C were higher in 2018. The WAI of Atlas, Dutchess, Pasto, and Summer Red WMF showed a continuous increase between 55 and 85 °C and was generally low compared to the WAI of other WMFs. A strong increase of the WAI, similar to what was observed in 2017, occurred at 95 °C. This resulted in medium-high WAIs for Atlas, Dutchess, and Pasto WMF (7.47–7.98 g/g, Table S1) but Summer Red WMF still had a low WAI at 95 °C (7.14 g/g, Table S1). Compared to the previous growing season (i.e., 2017), Atlas, Dutchess, Pasto, and Summer Red WMF of 2018 generally had higher WAIs between 75 and 95 °C.

The WMFs of 2019 showed a higher swelling behavior between 55 and 85 °C compared to the corresponding WMFs of previous years. Only at 95 °C were the WMFs of 2018 characterized by the highest WAIs (Table S1). In 2019, the group of Bastille, Faro, Oro de Valle, Titicaca, and Puno WMF was separated into three subgroups (Figure 2). Puno and Titicaca WMF no longer showed a continuous increase of WAI but the rate of increase decreased at 75 °C, resulting in medium-high WAI at 95 °C (7.31–7.66 g/g, Table S1). Puno and Titicaca WMF started off with a similar WAI at 55 °C but Titicaca had a higher WAI at 65 and 75 °C, and Puno at 85 and 95 °C. In contrast to previous years, the WAI of Oro de Valle WMF followed a different trend than the WAI of Bastille and Faro WMF. The WAI of Oro de Valle continuously increased between 55 and 85 °C but showed a deflection at 95 °C. This WMF was characterized by the highest WAI measured at 85 and 95 °C. Bastille and Faro WMF showed a low to medium-high swelling behavior between 55 and 85 °C, and up to 75 °C, the WAI of Bastille WMF was always lower compared to that of Faro. At 95 °C, both WMFs showed a similar and high WAI of 8.32 to 8.38 g/g (Table S1). While Jessie and Vikinga WMF showed a medium-high to high swelling behavior between 55 and 75 °C, its WAI was among the lowest at 85 and 95 °C. A deflection of the WAI at 95 °C was already observed for Jessie WMF in 2018, but was present for both WMFs in 2019. Furthermore, the WAI was improved less by increasing the temperature from 55 to 95 °C in 2019. In 2019, the strong increase in WAI between 85 and 95 °C was no longer observed for Atlas, Dutchess, Summer Red, and Zwarte WMF. In fact, the WAI of these WMFs followed a different trend compared to previous years, which separated the group into two subgroups. Atlas and Dutchess WMF showed a continuous increase in the WAI, with remarkably higher differences in WAI between both WMFs at 65 and 85 °C. The WAI of Rouge Marie, Summer Red, and Zwarte WMF showed a step-by-step increase, with the strongest improvements observed between 55 and 65 °C and between 75 and 85 °C. Rouge Marie and Summer Red WMF had a very similar swelling behavior between 75 and 95 °C and were considered WMF with a low WAI at these temperatures. Zwarte WMF had a higher WAI than Rouge Marie and Summer Red over the whole temperature.

As discussed above, quinoa WMFs showed a great diversity in their swelling behavior. Previous studies confirm that the swelling behavior of quinoa flour or starch is diverse and related this diversity to the variations in amylose content [9,13,26]. Lindeboom et al. compared quinoa lines with varying amylose content (3–20%) and noted a negative impact of the amylose content on the swelling power. Amylose is known for acting as a restraint to swelling, as it reinforces the internal structure of the starch granules [26]. The WMFs of Bastille, Faro, Oro de Valle, Puno, and Titicaca generally showed a strong improvement in the WAI as the temperature increased from 55 to 95 °C. Thus, this suggests a lower amylose content compared to WMFs, such as Jessie or Vikinga, which had a low WAI at 95 °C. The impact of the growing season suggests that the amylose content was also affected by the growing conditions. Nevertheless, Li et al. [9] noted that the swelling power of quinoa flour was considerably lower compared to that of its isolated starch. This indicates that other components, such as proteins and fat, could restrict the granule swelling as well. 

#### 2.2.3. Pasting Properties

**Figure 3:**
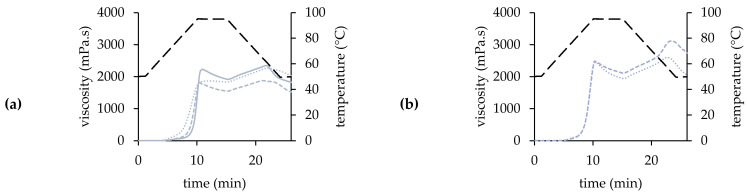

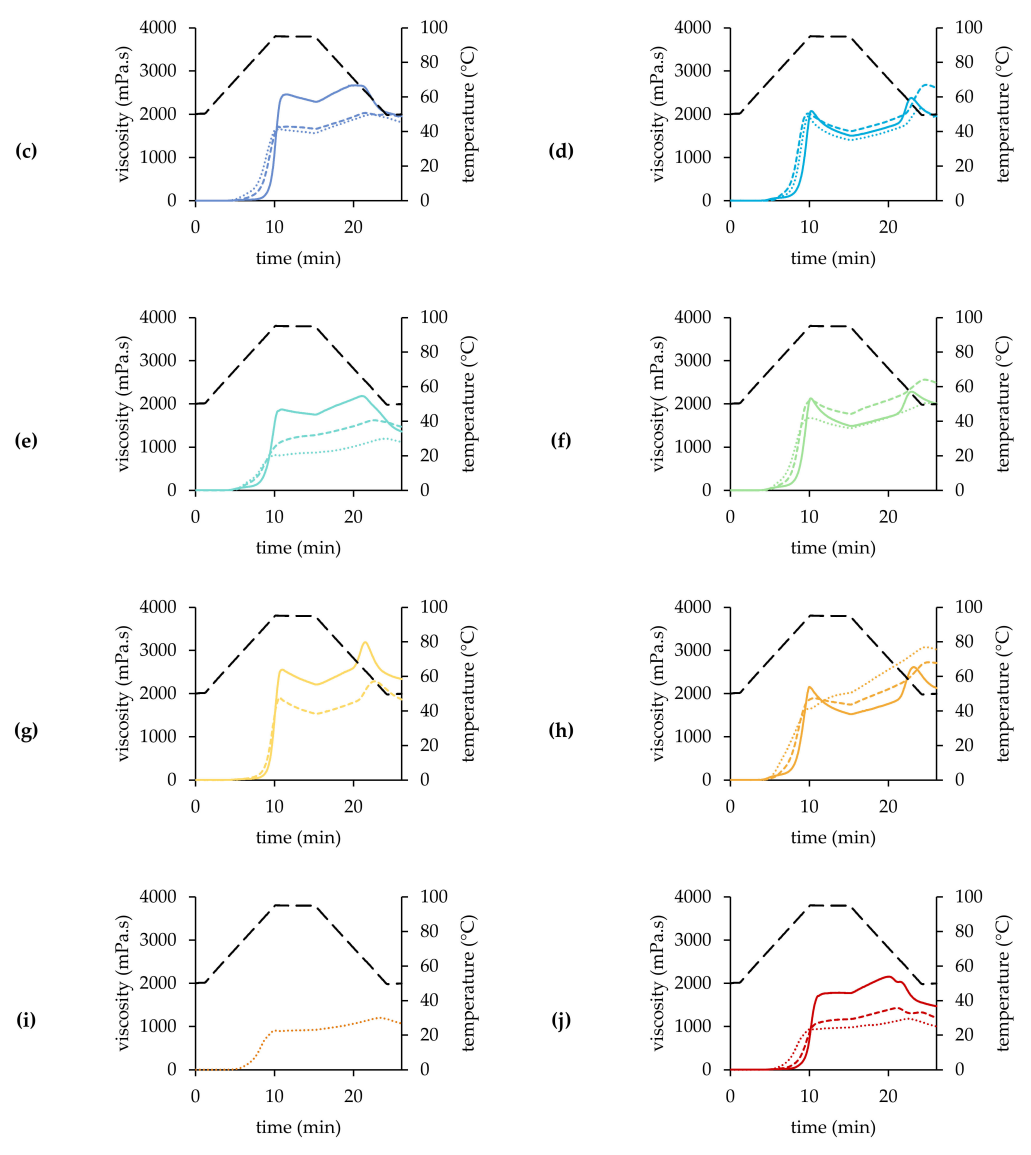

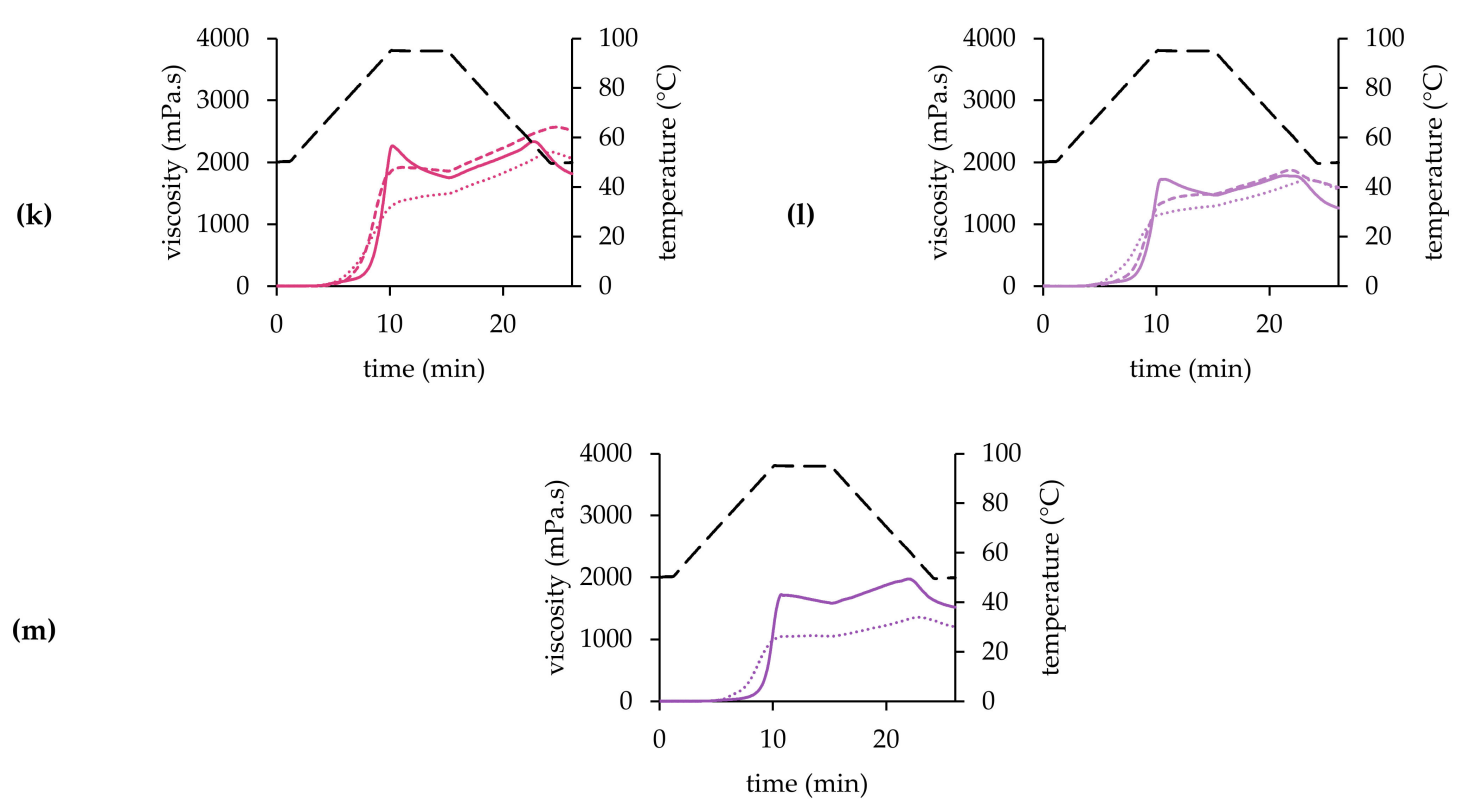
Pasting profile of 13 quinoa wholemeal flours ((**a**): Atlas, (**b**): Bastille, (**c**): Dutchess, (**d**): Faro, (**e**): Jessie, (**f**): Oro de Valle, (**g**): Pasto, (**h**): Puno, (**i**): Rouge Marie, (**j**)**:** Summer Red, (**k**): Titicaca, (**l**): Vikinga, (**m**): Zwarte) obtained from seeds grown under North-West European field conditions in 2017 (―), 2018 (- - -) and 2019 (· · ·) (temperature: ———, *n* = 3).

The pasting profile of a WMF enables the relation of the functionality of starch to its structural characteristics. It determines potential industrial applications in products, as these depend on the viscosity and thickening behavior of the WMF [1]. The pasting profiles (Figure 3) showed that the WMFs differed in pasting and viscosity characteristics, and were affected by a significant interaction between variety and year (*p* < 0.001, Table S4). The pasting profile was defined by the pasting temperature, peak viscosity and temperature, breakdown, second peak viscosity, final viscosity, and total setback. These parameters are summarized in Table S2 in Supplementary Materials. 

A certain temperature is required to launch the swelling process of the starch granules. This is the so-called pasting temperature, and is in fact the minimum temperature for starch gelatinization [1,27]. Over the years, the pasting temperature of the WMFs ranged between 57.70 and 67.11 °C (Table S2). Higher pasting temperatures have been reported for quinoa WMF (69.2 °C, [27]; 61.6–74.8 °C, [2]). The pasting temperature of Atlas, Bastille, Puno, and Vikinga WMF varied with the growing season. These WMFs generally had the highest pasting temperature in 2018, except for Puno. In 2018, a negative correlation between the pasting temperature and starch content (*r* = −0.640, *p* = 0.034) was observed. Tafadzwa et al. [23] confirmed that flours with a higher starch content had a lower pasting temperature. Oro de Valle and Titicaca were the only WMFs with a constant pasting temperature over the different years of cultivation. However, Dutchess, Jessie, Pasto, and Summer Red WMF showed a stable pasting temperature in 2017 and 2018, Faro and Zwarte WMF in 2017 and 2019. Pasting temperature correlated negatively with the WAI at 55 °C (*r* = –0.546, *p* = 0.001) and 65 °C (*r* = –0.650, *p* < 0.001). Thus, a high WAI indicates that the WMF is able to swell more freely, which results in a lower pasting temperature [20].

Peak viscosity occurs at an equilibrium point between granule swelling and polymer leaching. It is related to the water-binding capacity and the degree of swelling of the starch granules [9,27]. The peak viscosity can be indicative of end-product quality as well as the viscosity which is likely to be encountered by processes, such as mixing [2]. The peak viscosity showed a decreasing trend over the years of cultivation, and was affected by a significant variety × year interaction (*p* < 0.001, Table S4). The WMFs of 2017 had a peak viscosity between 1696 and 2560 mPa.s (Table S2) but higher values have been reported by Solaesa et al. (2690 mPa.s, [1]) and De Bock et al. (1418–2606 mPa.s, [20]). The high peak viscosities suggest a high degree of swelling of the starch granules during heating in 2017 [28]. However, WMFs of 2017 were characterized by low WAIs at 75 to 95 °C (Table S1), suggesting a low swelling behavior of these WMFs. This discrepancy might be related to the differences in measuring conditions (shear vs. no shear). Jessie, Summer Red, Vikinga, and Zwarte WMF developed the lowest peak viscosity that year, Pasto WMF the highest. Oro de Valle WMF showed a similar peak viscosity in 2017 and 2018, while the peak viscosity of other WMFs decreased (1030–2085 mPa.s, Table S2). Viscosity is directly related with the starch content, but the high starch levels did not result in the highest peak viscosity in 2018 [29]. The higher fat levels probably restricted the starch granule swelling due to complex formation with amylose upon heating, which reduced the viscosity of the WMFs of 2018 [29,30]. Jessie, Summer Red and Vikinga remained the WMFs with the lowest peak viscosity, while Bastille WMF reached the highest peak viscosity (2492 mPa.s) in 2018. Most WMFs had their lowest peak viscosity in 2019 (823–1959 mPa.s, Table S2), although the peak viscosity of Atlas (1871 mPa.s) and Bastille (2453 mPa.s) did not continue to decrease. Barak et al. [31] reported that flours with higher protein content reached lower peak viscosity. This might explain why the peak viscosities were generally lower compared to previous growing seasons in 2019 (*r* = −0.350, *p* = 0.042). Bastille remained the WMF with the highest peak viscosity, while the lowest viscosities were measured for Jessie, Rouge Marie, Summer Red, Titicaca, Vikinga, and Zwarte WMF.

The WMFs were characterized by a high peak temperature, which ranged between 93.69 and 95.22 °C (Table S2). The impact of growing season depended on the variety, indicating a significant variety × year interaction (*p* < 0.001, Table S4). The peak temperature of Faro and Puno WMF varied over the different growing seasons. Faro WMF obtained its lowest peak temperature in 2018, and differed significantly from all other WMFs of 2018. The growing season of 2019 resulted in the lowest peak temperature for Jessie, Oro de Valle, and Puno WMF. Jessie and Oro de Valle WMF were both characterized by a stable peak temperature in 2017 and 2018. The peak temperature of all other WMFs was not affected by the growing season. Peak temperature usually did not differ among varieties, except when the peak temperature was below 95 °C. Therefore, more variation in peak temperature was observed in 2019. The WMFs showed a high peak time, as peak viscosity was reached shortly before or during the 95 °C holding phase. Tiga et al. [27] attributed the high peak time of quinoa WMF to its high protein content, delaying the water absorption and swelling of the starch granules.

The breakdown measures the difference between the viscosity peak and the holding strength and is an indication of the stability against heat and shear [27]. The breakdown characterized the pasting profile of the WMF strongly, and was affected by a significant interaction between variety and year (*p* < 0.001, Table S4). All WMFs of 2017 were characterized by a positive breakdown (124–643 mPa.s, Table S2), except for Summer Red WMF. The high peak viscosities of these WMFs already suggested a high degree of starch granule swelling. The susceptibility to shear disruption increases as starch granules swell, which explains the high breakdown values in 2017 [13,28,32]. Puno and Oro de Valle WMF had the highest breakdown and showed the lowest stability against heat and shear. The lowest viscosity decreases were observed for Jessie, Dutchess, and Zwarte WMF. Summer Red WMF had a breakdown of −78 mPa.s, indicating that the viscosity continued to increase during the holding phase. This high stability against heat and shear is probably related to the low swelling behavior of Summer Red WMF (Table S1), as starch granules become increasingly susceptible to shear disruption as they swell [13]. The formation of amylose-lipid complexes may also play a role in the high shear stability, as Summer Red WMF contained high levels of fat [9]. The stability against heat and shear improved in 2018, except for Summer Red and Pasto WMF. The latter had a stable breakdown during the first two growing seasons. Jessie (−251 mPa.s) and Vikinga WMF (−187 mPa.s) also showed a negative breakdown in 2018 but the viscosity increase was much higher than the one observed for Summer Red WMF. The breakdown of other WMFs ranged between 65 and 410 mPa.s (Table S2), with the highest breakdown values measured for Pasto, Bastille and Faro WMF. The breakdown of Bastille, Faro, Jessie, Summer Red, and Vikinga WMF increased in 2019, but did not reach the same levels as in 2017. Bastille and Faro WMF were still characterized by the lowest stability against heat and shear. The breakdown of Atlas, Oro de Valle, Puno and Titicaca WMF continued to decrease in 2019, which resulted in negative breakdown values for Puno and Titicaca WMF. The latter showed the strongest viscosity increase among all WMFs with a negative breakdown. Puno and Titicaca WMF showed a medium-high swelling behavior in 2019, while their WAIs were relatively high compared to those of other WMFs in 2017 and 2018 (Table S1). The reduced swelling behavior in 2019 possibly promoted the shear and heat resistance during the holding phase [13]. The strong viscosity increase during the holding phase resulted in a unique pasting profile for Puno WMF of 2019 (Figure 3h), which significantly differed from that of previous growing seasons or other varieties. It is an indication that the water took longer to reach the starch granules and start the swelling process, and could be related to the increased protein content in 2019 [1]. However, Puno seeds were considerably smaller than the seeds of other varieties [19] and might show a different milling behavior due to the small seed size. Dziki et al. [33] concluded that small seeds were more difficult to reduce in size, resulting in a flour with a higher fraction of large particles. Solaesa et al. [1] concluded that the coarse fraction of quinoa flour showed no peak or valley in the pasting curve but a continuous increasing viscosity. Therefore, an increased share of large particles could possibly explain the unique pasting profile of Puno WMF in 2019. The breakdown value of Zwarte WMF was close to zero, indicating that the viscosity remained stable during the 95 °C holding phase. Aluwi et al. [2] also reported that the viscosity of Black WMF (possible origin for Zwarte [19]) remained relatively constant during the holding phase and increased shortly after.

The viscosity of the WMFs increased shortly after the holding phase. Due to the temperature decrease, starch chains associated and formed a more ordered structure, which increased the viscosity of the paste [34]. During this cooling phase, a second viscosity maximum, higher than the first peak, was observed in the pasting profile of all WMFs (Figure 3). The occurrence of a second peak has previously been described in other studies [1,2,20]. Over the years, this second peak viscosity ranged between 1183 and 3198 mPa.s (Table S2) and was closely related to the peak (*r* = 0.856, *p* < 0.001, Table S3) and final viscosity (*r* = 0.902, *p* < 0.001, Table S3). After the second peak, viscosity decreased and eventually reached final viscosity. This decrease might be attributed to the high protein levels in quinoa WMF, as the presence of proteins is known to reduce the resistance of gels to breakage [1]. The final viscosity indicates the ability of a WMF to form a viscous paste after cooking and cooling [27]. The final viscosity showed a wide variation over the different growing seasons and varieties, and varied between 1027 and 3047 mPa.s (Table S2). De Bock et al. [20] reported higher overall viscosities for WMFs from commercial quinoa seeds (1756–3274 mPa.s). The impact of the growing season strongly depended on the quinoa variety (variety × year: *p* < 0.001, Table S4). Most WMFs obtained the highest final viscosity in 2018. However, Pasto, Summer Red, and Zwarte WMF reached a higher final viscosity in 2017, Atlas and Puno WMF in 2019. The final viscosity of Dutchess, Faro, Oro de Valle, and Vikinga WMF did not vary every year, but showed a two-year stability over the experimental period from 2017 to 2019. Over the years, Jessie, Rouge Marie, Summer Red, and Zwarte were characterized as the WMFs with the lowest final viscosity. Puno and Bastille WMFs usually reached the highest final viscosities among all WMFs.

The total setback indicates the recovery of viscosity during cooling of the heated WMF suspension. It represents the tendency of the paste to retrograde and amylose to re-associate [11,27]. Atlas, Dutchess, Jessie, Summer Red, Vikinga, and Zwarte WMF were characterized by a negative setback in 2017, which ranged between −43 and −350 mPa.s (Table S2). Negative setback values for quinoa WMF have been reported before [35]. The negative values were related to a strong viscosity decrease after the second peak viscosity, which eventually resulted in a final viscosity lower than the holding strength. The setback of the other WMFs ranged between 108 and 654 mPa.s (Table S2), with the highest setback measured for Puno WMF. In 2018, the total setback ranged between 19 and 1037 mPa.s (Table S2), and had significantly increased compared to the previous growing season. This increase could be related to the reduced protein levels in the WMFs of 2018 [31]. Faro and Puno WMF showed a high setback, while the setback was low for Atlas and Summer Red WMF. The total setback of Atlas, Vikinga, and Zwarte WMF continued to increase in 2019, while it remained stable for Dutchess, Jessie, Puno, and Summer Red WMF. Summer Red WMF had the lowest setback (46 mPa.s), Puno WMF the highest (1030 mPa.s).

### 2.3. Principal Components Analysis and Hierarchical Cluster Analysis

**Figure 4:**
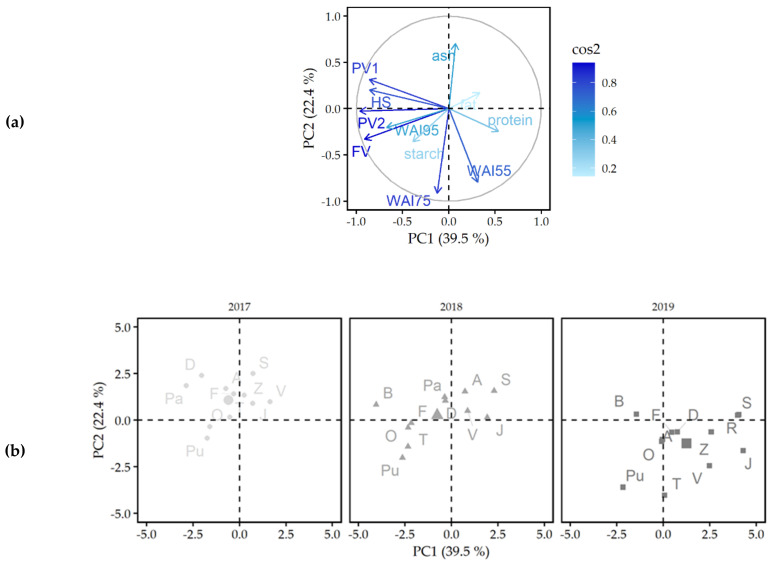
Principal components analysis (PCA; (**a**): variables plot, (**b**): score plot) of physicochemical properties of 13 quinoa wholemeal flours (A: Atlas, B: Bastille, D: Dutchess, F: Faro, J: Jessie, O: Oro de Valle, Pa: Pasto, Pu: Puno, R: Rouge Marie, S: Summer Red, T: Titicaca, V: Vikinga, Z: Zwarte) obtained from seeds grown under North-West European field conditions in 2017 (●), 2018 (▲) and 2019 (■). WAC: water absorption capacity, WAI: water absorption index, PV1: first peak viscosity, HS: holding strength, BD: breakdown, PV2: second peak viscosity, FV: final viscosity, TSB: total setback.

For a more comprehensive understanding of the variation among WMFs, a PCA was performed on the macronutrient composition (starch, protein, fat, ash), WAIs (55, 75 and 95 °C) and pasting properties (first peak viscosity, breakdown, second peak viscosity, final viscosity and total setback). The variables plot of the first two PCs is visualized in Figure 4. The first and second PC accounted for 39.5 and 22.4% of the variance, respectively. Together, the first two PCs represented 61.9% of the total variance in the data. The first PC was mainly defined by the first and second peak viscosity, holding strength and final viscosity. These were all properties related to viscosity and closely inter-correlated (Table S3), as also concluded by Li et al. [9]. The second PC was defined by the WAI at 55 and 75 °C. A positive correlation between these parameters was observed (*r* = 0.627, *p* < 0.001, Table S3). A higher WAI at 55 °C indicated a higher water uptake by the WMF. A better hydration of the WMF possibly improved the starch gelatinization and, therefore, resulted in a higher WAI at 75 °C. 

The score plot (Figure 4) showed that the grouping of the WMFs was generally consistent over different growing seasons. Within a given growing season, the WMFs were closely grouped with exception of some varieties. In 2017, Oro de Valle and Puno were separated from the other WMFs due to the low ash content and high WAI at 75 °C. The same WMFs, together with Bastille, Faro, and Titicaca WMF, showed a higher score on PC1 compared to other WMFs of 2018. These high scores on PC1 were attributed to the high WAI at 95 °C, high second peak and final viscosity of these WMFs. The high swelling behavior at 75 °C separated Puno and Titicaca WMF from the other WMFs of 2019. The growing season caused a distinct shift in the scores of the WMFs. The WMFs of 2017 and 2018 were located on the upper side from the central axis of PC2, while most WMFs of 2019 were grouped in the lower right quadrant of the score plot. The WMFs of 2019 showed higher swelling behavior at 55 and 75 °C but developed lower pasting viscosities, which explains the positive scores on PC2 and the negative scores on PC1.

**Figure 5:**
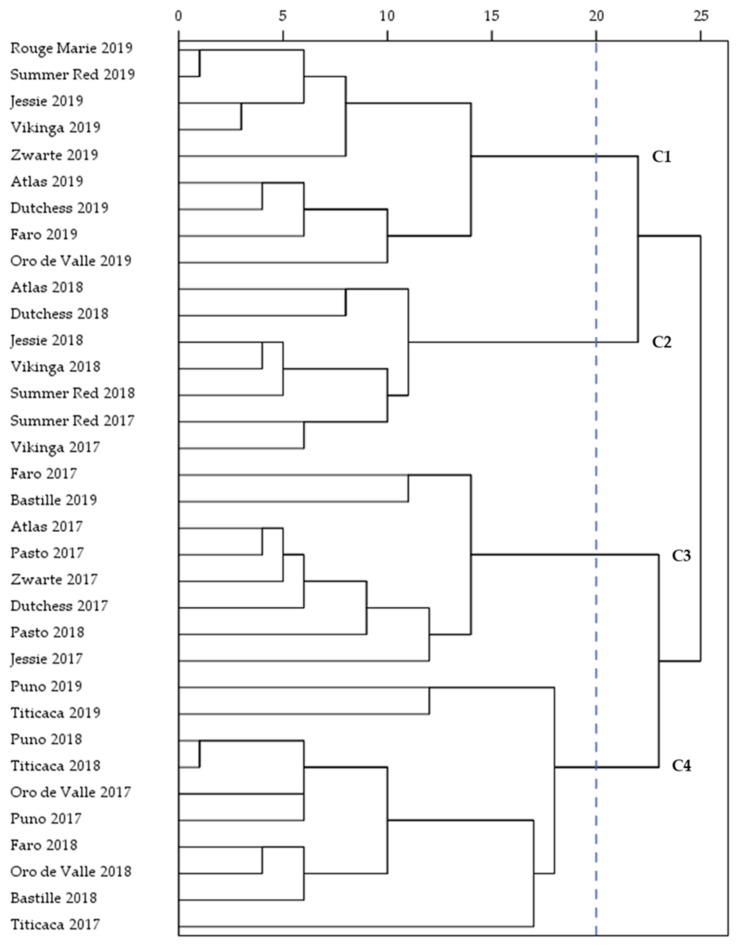
Dendrogram of the four clusters (C1–C4) based on hierarchical clustering.

The macronutrient composition (starch, protein, fat, ash), WAIs (55, 75 and 95 °C), and pasting properties (first peak viscosity, breakdown, second peak viscosity, final viscosity and total setback) were further analyzed by HCA and the corresponding results were in good agreement with the PCA results. A total of four clusters were identified based on these parameters and the dendrogram of the hierarchical clustering is illustrated in Figure 5. The clusters (C1-C4) consisted of *n* = 9 WMFs, *n* = 7 WMFs, *n* = 8 WMFs and *n* = 10 WMFs, respectively. The first cluster grouped the WMFs which were mainly located in the lower right quadrant of the score plot of the PCA (Figure 4). This means that the cluster included all WMFs of 2019, except for Bastille, Puno, and Titicaca WMF. These WMFs were characterized by the highest WAI at 55 and 75 °C and generally showed lower peak viscosity and holding strength than the corresponding WMFs of 2017 and 2018. The second cluster grouped the WMFs with the lowest viscosity parameters in 2017 and 2018. This cluster included Summer Red and Vikinga WMF of 2017 and Atlas, Dutchess, Jessie, Summer Red, and Vikinga WMF of 2018. These WMFs were all characterized by a high score on PC1 (Figure 4). The third cluster mainly consisted of WMFs of 2017, Atlas, Dutchess, Faro, Jessie, Pasto, and Zwarte WMF, respectively. Overall, WMFs of 2017 were characterized by the highest first peak viscosity, the least stability against heat and shear and a low tendency to retrograde. Furthermore, Pasto WMF of 2018 and Bastille WMF of 2019 were included in cluster 3. The WMFs of the third cluster were generally located in the upper left quadrant of the score plot (Figure 4). The score plot showed that certain WMFs differentiated more from others despite the fact that the seeds were cultivated under the same growing conditions (Figure 4). These WMFs were grouped in the fourth cluster. This cluster included Oro de Valle, Puno, and Titicaca WMF of 2017, Bastille, Faro, Oro de Valle, Puno, and Titicaca WMF of 2018 and Puno and Titicaca WMF of 2019. The fact that all Puno and Titicaca WMFs were grouped within the same cluster indicates that the variety had a larger impact on the physicochemical properties of these WMFs than the growing season. 

## 3. Material and Methods

### 3.1. Quinoa Wholemeal Flours

Ten quinoa (*Chenopodium quinoa* Willd.) varieties were purchased at commercial companies (Gilbel sprl, Saint-Georges-Sur-Meuse, Belgium (previously a sublicensee of Radicle Crops, Wageningen, The Netherlands) and Quinoa Quality ApS, (Regstrup, Denmark) and three Farm Original varieties were obtained from De Nieuwe Tuin (De Klinge, Belgium). These 13 quinoa varieties were used for a comparative variety testing under Belgium field conditions during three consecutive growing seasons (2017–2019). Information on the variety origins and crop cultivation is described in detail in a previous work [19]. For chemical and physicochemical analyses, seeds were milled to WMF by a Hammertec mill (mesh size: 0.8 mm) (Foss, Hilleroed, Denmark).

### 3.2. Chemical Composition

Moisture (g/100 g) and ash (g/100 g dm) content were determined according to ICC methods no. 110 and 104/1, respectively. Total starch content (g/100 g dm) was analyzed as described by Englyst et al. [36]. The nitrogen content was determined by the use of a VarioMax C/N (Elementar Analysesystemen, Langenselbold, Germany) and converted to protein content (g/100 g dm) using a conversion factor of 6.25 [37]. Fat content (g/100 g dm) was determined by Soxhlet extraction with prior acid hydrolysis (ISO 6491). Lastly, crude fiber was calculated by the difference.

### 3.3. Physicochemical Properties

WAC and WAI of the WMFs were determined as described by De Bock et al. [20]. In brief, an aqueous suspension of 0.075 g WMF in 1.5 mL distilled water was shaken for 30 min at 1000 rpm in a ThermoMixer C (Eppendorf, Hamburg, Germany). For the determination of the WAC, the shaking temperature was 21 °C. The WAI was determined at a shaking temperature of 55, 65, 75, 85, and 95 °C. Subsequently, the suspension was centrifuged for 20 min at 8000× *g* and 21 °C. The supernatant was decanted and the sediment was weighed. WAC (g/g) was expressed as the amount of water absorbed by the WMF. WAI (g/g) was calculated as the weight of sediment per gram of WMF used.

Pasting properties of the WMFs were determined using a Rheometer MCR 102 (Anton Paar GmbH, Graz, Austria). Measurement was performed using 2.8 g WMF (based on 14% moisture) dispersed in 20 mL of distilled water. During the pre-shear phase, the suspension was heated to 50 °C while stirred at 960 rpm. The rotation speed was 160 rpm for the remainder of the test. The temperature was initially maintained at 50 °C for 1 min and then raised to 95 °C at a constant rate of 5 °C per min, held at 95 °C for 5 min, cooled to 50 °C at the same rate and finally held at 50 °C for 2 min. The parameters recorded were the pasting temperature (°C, temperature of initial viscosity increase), first peak viscosity (mPa.s, viscosity maximum during heating and/or holding phase), peak temperature (°C, temperature to reach first peak viscosity), holding strength (mPa.s, minimum viscosity at 95 °C), breakdown (mPa.s, difference between first peak viscosity and holding strength), second peak viscosity (mPa.s, viscosity maximum during cooling), final viscosity (mPa.s, viscosity at test finish) and total setback (mPa.s, difference between final viscosity and first peak viscosity) [38].

### 3.4. Statistical Analysis

The lme4 package in R (version 4.0.2, R Core Team, Vienna, Austria) was used to fit a linear model [39]. The fixed effects were variety and year, and the following model (1) was considered:

Y = Variety + Year + Variety × Year(1)

where ‘Y’ is the response variable, ‘Variety’ is the fixed effect of the variety, ‘Year’ is the fixed effect of the year and ‘Variety × Year’ is the interaction effect between variety and year. Then, an analysis of variance (ANOVA) was performed (Table S4) and a pairwise comparison between varieties and years was made with the Tukey test.

For a more comprehensive understanding of the variation among WMFs, a PCA was performed on the macronutrient composition (starch, protein, fat, ash), WAIs (55, 75 and 95 °C) and pasting properties (first peak viscosity, breakdown, second peak viscosity, final viscosity, and total setback). PCA was performed using the built-in R function ‘prcomp’ (R Foundation for Statistical Computing, Vienna, Austria). The graphs were made by the library ‘factoextra’ in combination with the ggplot2-package of R. Additionally, a Pearson correlation test was conducted to study the correlations between the physicochemical parameters more deeply (Table S3). The macronutrient composition (starch, protein, fat, ash), WAIs (55, 75 and 95 °C) and pasting properties (first peak viscosity, breakdown, second peak viscosity, final viscosity and total setback) were used to perform an HCA to group the WMFs with similar properties. HCA was performed with SPSS Statistics 27 (SPSS Inc., Chicago, IL, USA) and the unweighted paired-group method with arithmetic mean was used. Distances among clusters were computed using Pearson correlation coefficients.

## 4. Conclusions

A comparative study was performed on ten European and three Farm Original (USA) quinoa varieties cultivated under North-West European field conditions during three consecutive growing seasons (2017–2019). Within the present study the quinoa seeds were milled to WMF, and their physicochemical properties were evaluated, more precisely the WAC, the WAI upon heating and the pasting properties. The results revealed large differences in physicochemical properties among quinoa WMFs and depended on the variety and the growing season. The WMFs of 2017 had the lowest WAIs at temperatures above 55 °C. The high peak viscosity of these WMFs was followed by a strong viscosity breakdown and the paste showed a low tendency to retrograde. The WAIs of the WMFs significantly increased in 2018. Moreover, the WMFs of 2018 were characterized by the highest WAI at 95 °C. This high swelling behavior was probably related to the high starch content of these WMFs. The pasting profiles were characterized by lower peak viscosities, improved heat and shear resistance, and higher setback values. The WMFs of 2019 contained the highest levels of protein, which improved the ability of these WMFs to absorb and retain water. This was reflected in the high WAC and WAIs at 55 and 65 °C, but resulted in more physical competition for water between proteins and starch. This probably restricted the starch granule swelling, which explains the low peak viscosity and holding strength of the WMFs of 2019. The impact of variety was less consistent than the effect of the growing season. However, certain trends were observed over the years. Jessie and Titicaca WMF had a high WAC while Oro de Valle WMF showed a high swelling behavior upon heating. Jessie, Summer Red, Rouge Marie, Vikinga, and Zwarte were WMFs characterized by a low swelling behavior. These WMFs developed low viscosities and had a high resistance to shear and heat. The pasting profile of Bastille WMF was generally characterized by a high peak and final viscosity and, together with Faro WMF, showed a high breakdown. Puno WMF developed a high final viscosity upon cooling, indicating a high tendency to retrograde.

The wide variation in physicochemical properties suggest that the potential food applications of WMFs depend on the quinoa variety. However, the growing conditions may significantly alter the macronutrient composition of the seeds, thereby affecting the physicochemical properties of the WMFs and eventually the end-product quality. Nevertheless, variations in the macronutrient composition only partially explained the differences in physicochemical properties. The impact of other components such as fiber, amylose/amylopectin, and damaged starch on the physicochemical properties of these quinoa WMFs might be the subject of further research. 

## Figures and Tables

**Table 1:** Chemical composition (g/100 g dm) of 13 quinoa varieties grown under North-West European field conditions in 2017, 2018, and 2019.

Year	Variety	Protein (g/100 g dm) ^1^	Fat (g/100 g dm) ^1^	Starch (g/100 g dm) ^1^	Ash (g/100 g dm) ^1^
2017	Atlas	16.6 ± 0.2 ^fA^	6.41 ± 0.04 ^cA^	58.1 ± 0.6 ^dB^	3.46 ± 0.02 ^gC^
	Bastille	−	−	−	−
	Dutchess	13.8 ± 0.0 ^cA^	7.03 ± 0.07 ^eA^	59.6 ± 0.5 ^eB^	3.35 ± 0.01 ^fB^
	Faro	14.9 ± 0.3 ^dB^	7.33 ± 0.09 ^fA^	53.4 ± 0.7 ^aB^	3.32 ± 0.02 ^efB^
	Jessie	16.0 ± 0.2 ^eB^	6.70 ± 0.04 ^dA^	54.9 ± 0.7 ^bA^	3.12 ± 0.02 ^dC^
	Oro de Valle	12.1 ± 0.2 ^aA^	6.18 ± 0.03 ^cA^	67.2 ± 1.2 ^gC^	2.86 ± 0.03 ^bA^
	Pasto	15.5 ± 0.3 ^eA^	5.42 ± 0.05 ^aA^	56.5 ± 0.5 ^cA^	3.47 ± 0.02 ^gA^
	Puno	13.9 ± 0.2 ^cB^	5.65 ± 0.02 ^abA^	61.7 ± 0.4 ^fB^	2.37 ± 0.02 ^aA^
	Rouge Marie	−	−	−	−
	Summer Red	13.9 ± 0.2 ^cB^	8.54 ± 0.08 ^iC^	58.0 ± 0.2 ^dC^	3.29 ± 0.01 ^eB^
	Titicaca	14.8 ± 0.1 ^dB^	7.61 ± 0.08 ^gB^	60.7 ± 0.8 ^fA^	2.84 ± 0.01 ^bAB^
	Vikinga	14.9 ± 0.4 ^dB^	8.04 ± 0.08 ^hB^	57.8 ± 0.7 ^dA^	3.02 ± 0.03 ^cB^
	Zwarte	13.0 ± 0.3 ^bA^	5.70 ± 0.03 ^bA^	55.2 ± 0.7 ^bA^	3.16 ± 0.03 ^dA^
2018	Atlas	16.4 ± 0.1 ^fA^	7.78 ± 0.03 ^eC^	57.5 ± 0.5 ^aB^	3.30 ± 0.02 ^cB^
	Bastille	13.1 ± 0.2 ^bA^	7.34 ± 0.01 ^dA^	64.0 ± 0.3 ^eB^	3.09 ± 0.03 ^bB^
	Dutchess	14.4 ± 0.2 ^eB^	7.75 ± 0.04 ^eB^	61.4 ± 0.4 ^cC^	3.32 ± 0.02 ^cB^
	Faro	13.2 ± 0.2 ^bA^	8.09 ± 0.08 ^fC^	58.2 ± 0.4 ^abC^	3.07 ± 0.01 ^bA^
	Jessie	13.7 ± 0.1 ^cdA^	7.93 ± 0.05 ^efB^	59.1 ± 0.4 ^bC^	3.08 ± 0.01 ^bB^
	Oro de Valle	12.2 ± 0.1 ^aA^	7.73 ± 0.06 ^eC^	62.5 ± 0.6 ^dB^	3.08 ± 0.01 ^bC^
	Pasto	16.0 ± 0.2 ^fB^	6.37 ± 0.04 ^aB^	59.0 ± 0.6 ^bB^	3.60 ± 0.02 ^dB^
	Puno	12.5 ± 0.2 ^aA^	6.67 ± 0.03 ^bC^	63.7 ± 0.3 ^eC^	2.90 ± 0.02 ^aC^
	Rouge Marie	−	−	−	−
	Summer Red	13.3 ± 0.1 ^bcA^	8.18 ± 0.01 ^fB^	57.3 ± 0.3 ^aB^	3.27 ± 0.03 ^cB^
	Titicaca	13.2 ± 0.1 ^bA^	7.08 ± 0.03 ^cA^	63.9 ± 0.7 ^eB^	2.88 ± 0.01 ^aB^
	Vikinga	13.8 ± 0.4 ^dA^	8.50 ± 0.09 ^gC^	63.6 ± 0.2 ^eC^	3.08 ± 0.03 ^bC^
	Zwarte	−	−	−	−
2019	Atlas	18.5 ± 0.0 ^fgB^	7.20 ± 0.14 ^dB^	54.5 ± 0.4 ^cA^	3.03 ± 0.02 ^cdA^
	Bastille	16.6 ± 0.0 ^bcB^	7.36 ± 0.19 ^deA^	52.3 ± 0.5 ^bA^	3.01 ± 0.03 ^cdA^
	Dutchess	18.4 ± 0.1 ^fgC^	7.17 ± 0.04 ^dA^	54.8 ± 0.2 ^cdA^	3.21 ± 0.01 ^fA^
	Faro	17.6 ± 0.1 ^dC^	7.61 ± 0.12 ^efB^	50.5 ± 0.4 ^aA^	3.09 ± 0.01 ^deA^
	Jessie	18.8 ± 0.0 ^gC^	6.70 ± 0.24 ^cA^	55.6 ± 1.3 ^deB^	2.84 ± 0.03 ^aC^
	Oro de Valle	16.2 ± 0.0 ^abB^	7.22 ± 0.07 ^dB^	54.7 ± 0.8 ^cdA^	2.91 ± 0.02 ^bB^
	Pasto	−	−	−	−
	Puno	17.0 ± 0.1 ^cC^	5.88 ± 0.24 ^aB^	59.4 ± 1.1 ^fA^	2.83 ± 0.01 ^aB^
	Rouge Marie	17.8 ± 0.1 ^deA^	7.65 ± 0.08 ^fA^	53.9 ± 0.2 ^cA^	3.10 ± 0.02 ^eA^
	Summer Red	18.1 ± 0.2 ^efC^	7.75 ± 0.09 ^fA^	54.5 ± 0.4 ^cA^	3.20 ± 0.01 ^fA^
	Titicaca	16.0 ± 0.1 ^aC^	7.25 ± 0.03 ^dA^	72.5 ± 0.9 ^hC^	2.83 ± 0.02 ^aA^
	Vikinga	17.0 ± 0.1 ^cC^	7.71 ± 0.04 ^fA^	60.8 ± 0.5 ^gB^	2.82 ± 0.02 ^aA^
	Zwarte	16.0 ± 0.1 ^aB^	6.36 ± 0.08 ^bB^	56.2 ± 0.2 ^eB^	3.29 ± 0.04 ^gB^

^1^ Within years, average values followed by the same lowercase letter are not significantly different (*p* > 0.05). Capital letters compare the three years for the same variety; average values followed by the same letter are not significantly different (*p* > 0.05).

## Data Availability

The data presented in this study are available in the article and Supplementary Materials.

## Supplementary Materials

The following are available online at https://www.mdpi.com/article/10.3390/plants11030265/s1, Figure S1: Chemical composition of thirteen quinoa varieties ((**a**): Atlas, (**b**): Bastille, (**c**): Dutchess, (**d**): Faro, (**e**): Jessie, (**f**): Oro de Valle, (**g**): Pasto, (**h**): Puno, (**i**): Rouge Marie, (j): Summer Red, (**k**): Titicaca, (**l**): Vikinga, (**m**): Zwarte) grown under North-West European field conditions in 2017 (●), 2018 (●) and 2019 (●). Values are presented as the mean relative to the three-year mean., Table S1: Water absorption index at 55, 65, 75, 85, and 95 °C (WAI, g/g) of thirteen quinoa wholemeal flours obtained from seeds grown under North-West European field conditions in 2017, 2018, and 2019 (*n* = 3), Table S2: Pasting parameters of thirteen quinoa whole-meal flours obtained from seeds grown under North-West European field conditions in 2017, 2018 and 2019 (*n* = 3), Table S3: Pearson correlation analysis between physicochemical properties, Table S4: Results of ANOVA (factors, *p* value).
