# Supplementary material for: Physicochemical Characterization of Thirteen Quinoa (Chenopodium quinoa Willd.) Varieties Grown in North-West Europe—Part II
Source: Plants (Basel). 2022 Jan 19;11(3):265. doi: 10.3390/plants11030265 (PMC8838886; doi:10.3390/plants11030265)
Supplement: Supplementary file 1 [file plants-11-00265-s001.zip › plants-1560412-supplementary.pdf]

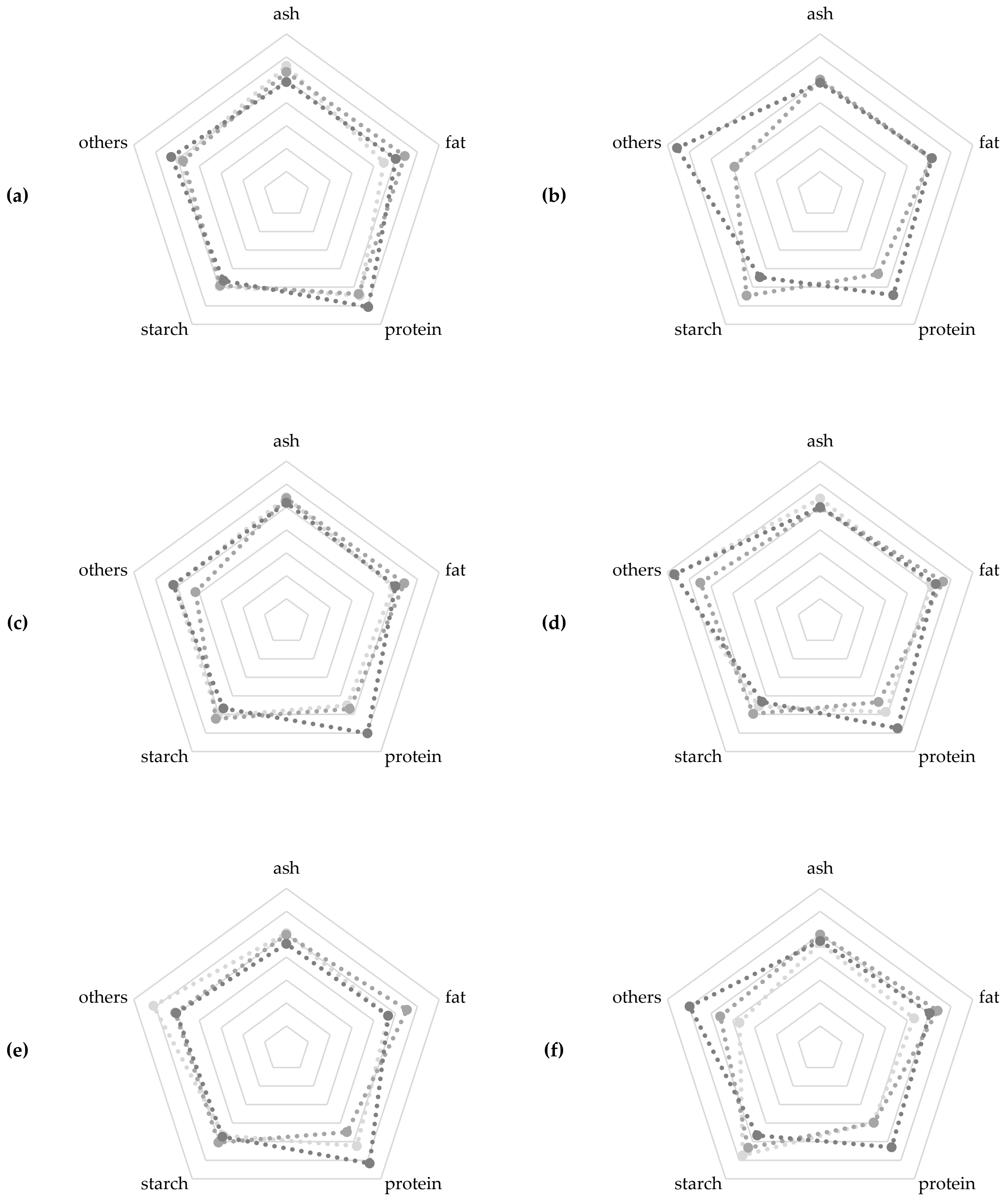
(a)
ash
fat
protein
starch
others
(b)
ash
fat
protein
starch
others
(c)
ash
fat
protein
starch
others
(d)
ash
fat
protein
starch
others
(e)
ash
fat
protein
starch
others
(f)
ash
fat
protein
starch
others

(g)

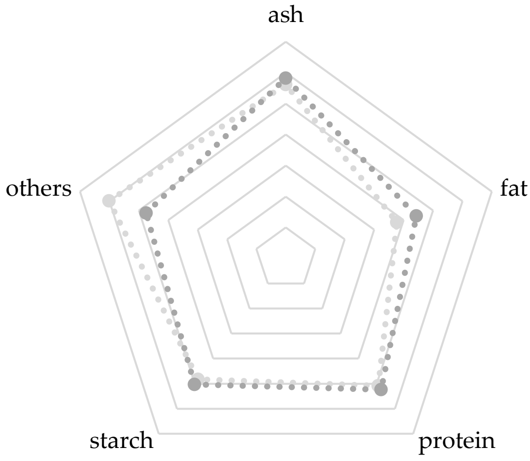
ash
fat
protein
starch
others

(h)

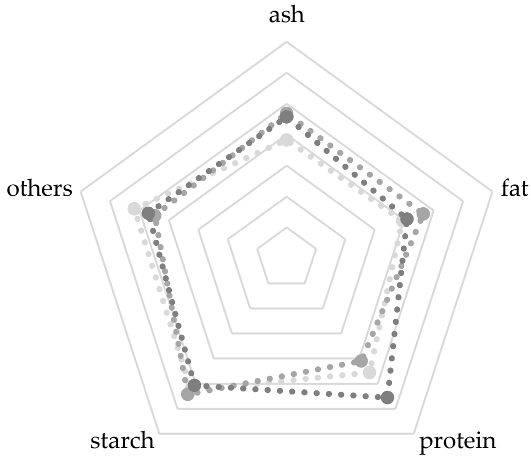
ash
fat
protein
starch
others

(i)

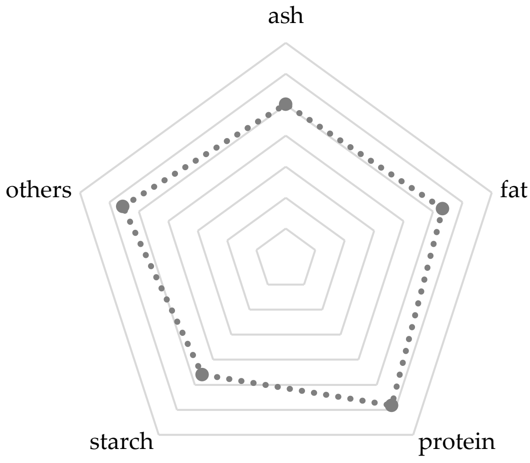
ash
fat
protein
starch
others

(j)

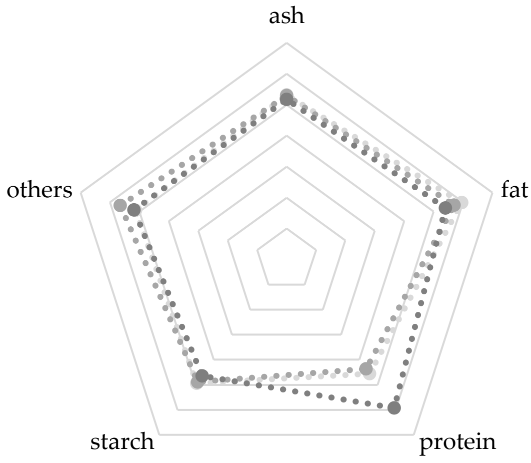
ash
fat
protein
starch
others

(k)

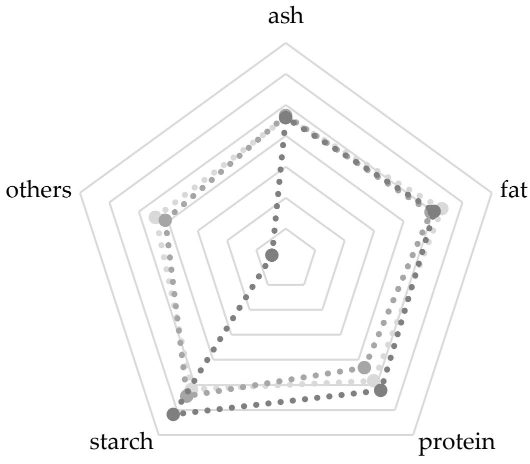
ash
fat
protein
starch
others

(l)

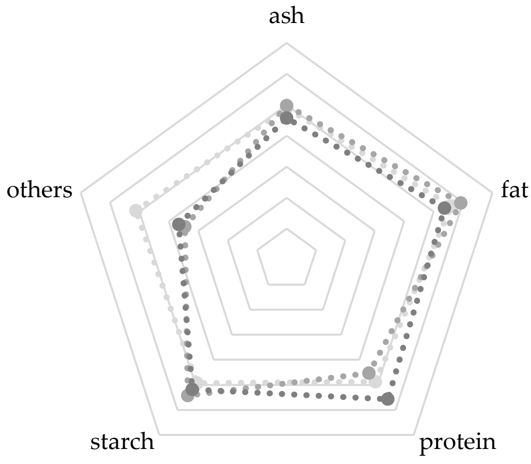
ash
fat
protein
starch
others

(m)

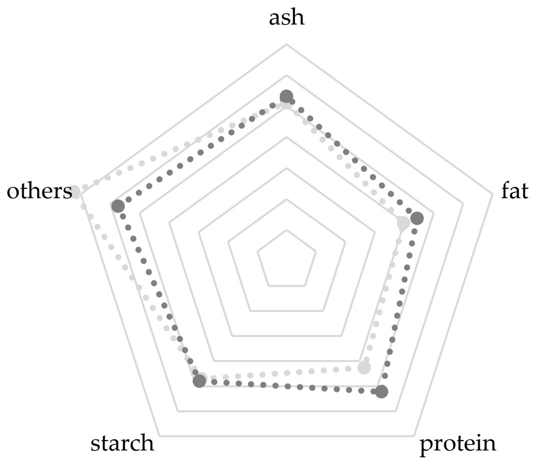

Figure S1. Chemical composition of thirteen quinoa varieties ((**a**): Atlas, (**b**): Bastille, (**c**): Dutchess, (**d**): Faro, (**e**): Jessie, (**f**): Oro de Valle, (**g**): Pasto, (**h**): Puno, (**i**): Rouge Marie, (**j**): Summer Red, (**k**): Titicaca, (**l**): Vikinga, (**m**): Zwarte) grown under North-West European field conditions in 2017 (●), 2018 (●) and 2019 (●). Values are presented as the mean relative to the three-year mean.

Table S1. Water absorption index at 55, 65, 75, 85 and 95 °C (WAI, g/g) of thirteen quinoa wholemeal flours obtained from seeds grown under North-West European field conditions in 2017, 2018 and 2019 (n = 3).

**Year**	**Variety**	**WAI 55 °C (g/g)** [1,2]	**WAI 65 °C (g/g)** [1,2]	**WAI 75 °C (g/g)** [1,2]	**WAI 85 °C (g/g)** [1,2]	**WAI 95 °C (g/g)** [1,2]
2017	Atlas	2.42 ± 0.03 ^{abA}	3.54 ± 0.03 ^{bcdB}	4.14 ± 0.02 ^{deB}	4.52 ± 0.05 ^{cA}	6.94 ± 0.13 ^{dA}
	Bastille	–	–	–	–	–
	Dutchess	2.29 ± 0.03 ^{aA}	3.42 ± 0.01 ^{bA}	3.75 ± 0.03 ^{bA}	4.30 ± 0.07 ^{bA}	6.68 ± 0.08 ^{bcA}
	Faro	2.35 ± 0.02 ^{abA}	3.67 ± 0.05 ^{defB}	4.48 ± 0.04 ^{fgA}	5.62 ± 0.03 ^{eA}	7.25 ± 0.07 ^{eA}
	Jessie	2.77 ± 0.04 ^{efB}	3.47 ± 0.04 ^{bcA}	4.23 ± 0.08 ^{eA}	5.12 ± 0.04 ^{dA}	6.16 ± 0.10 ^{aB}
	Oro de Valle	2.59 ± 0.02 ^{cdB}	3.84 ± 0.04 ^{fgA}	4.39 ± 0.03 ^{fA}	5.92 ± 0.09 ^{fA}	7.76 ± 0.01 ^{fA}
	Pasto	2.47 ± 0.07 ^{bcA}	3.59 ± 0.10 ^{cdeB}	3.89 ± 0.03 ^{cA}	4.33 ± 0.08 ^{bA}	7.25 ± 0.08 ^{eA}
	Puno	2.29 ± 0.09 ^{aA}	3.95 ± 0.08 ^{gA}	4.57 ± 0.06 ^{gA}	6.04 ± 0.04 ^{fA}	7.32 ± 0.04 ^{eA}
	Rouge Marie	–	–	–	–	–
	Summer Red	2.30 ± 0.06 ^{aA}	3.13 ± 0.02 ^{aA}	3.54 ± 0.02 ^{aA}	4.04 ± 0.07 ^{aA}	6.00 ± 0.06 ^{aA}
	Titicaca	2.87 ± 0.03 ^{fB}	3.74 ± 0.05 ^{efA}	4.11 ± 0.03 ^{deA}	5.62 ± 0.03 ^{eA}	6.65 ± 0.02 ^{bA}
	Vikinga	2.71 ± 0.08 ^{deB}	3.53 ± 0.06 ^{bcdA}	4.03 ± 0.04 ^{dA}	5.19 ± 0.04 ^{dA}	6.00 ± 0.04 ^{aA}
	Zwarte	2.60 ± 0.05 ^{cdA}	3.51 ± 0.01 ^{bcdA}	3.81 ± 0.02 ^{bcA}	4.32 ± 0.02 ^{bA}	6.85 ± 0.05 ^{cdA}
2018	Atlas	2.35 ± 0.02 ^{bA}	3.27 ± 0.07 ^{aA}	4.03 ± 0.04 ^{bA}	4.85 ± 0.05 ^{aB}	7.47 ± 0.09 ^{cB}
	Bastille	2.04 ± 0.03 ^{aA}	3.33 ± 0.02 ^{aA}	4.44 ± 0.03 ^{dA}	6.28 ± 0.09 ^{eA}	8.85 ± 0.04 ^{fB}
	Dutchess	2.42 ± 0.05 ^{bcB}	3.35 ± 0.02 ^{abA}	4.29 ± 0.04 ^{cB}	5.35 ± 0.01 ^{cB}	7.84 ± 0.04 ^{dB}
	Faro	2.67 ± 0.02 ^{efB}	3.29 ± 0.09 ^{aA}	4.65 ± 0.02 ^{eB}	7.35 ± 0.09 ^{gC}	9.48 ± 0.06 ^{gC}
	Jessie	2.57 ± 0.07 ^{deA}	3.39 ± 0.01 ^{abA}	4.77 ± 0.07 ^{eB}	6.25 ± 0.01 ^{eC}	6.48 ± 0.05 ^{aC}
	Oro de Valle	2.72 ± 0.02 ^{fgC}	3.98 ± 0.05 ^{dB}	4.97 ± 0.01 ^{fB}	7.59 ± 0.02 ^{hB}	8.73 ± 0.04 ^{fC}
	Pasto	2.73 ± 0.04 ^{fgB}	3.24 ± 0.04 ^{aA}	4.00 ± 0.08 ^{abB}	5.04 ± 0.01 ^{bB}	7.98 ± 0.02 ^{dB}
	Puno	2.85 ± 0.03 ^{gB}	4.16 ± 0.02 ^{eB}	5.90 ± 0.04 ^{hB}	6.93 ± 0.08 ^{fB}	8.47 ± 0.05 ^{eC}
	Rouge Marie	–	–	–	–	–
	Summer Red	2.44 ± 0.02 ^{bcdB}	3.69 ± 0.09 ^{cB}	3.87 ± 0.03 ^{aB}	4.81 ± 0.10 ^{aB}	7.14 ± 0.05 ^{bC}
	Titicaca	2.68 ± 0.04 ^{efA}	4.58 ± 0.06 ^{fB}	5.56 ± 0.05 ^{gB}	7.36 ± 0.01 ^{gC}	8.35 ± 0.02 ^{eC}
	Vikinga	2.53 ± 0.01 ^{cdA}	3.51 ± 0.01 ^{bA}	4.27 ± 0.06 ^{cB}	6.05 ± 0.05 ^{dB}	7.14 ± 0.04 ^{bB}
	Zwarte	–	–	–	–	–
2019	Atlas	2.99 ± 0.04 ^{defB}	4.76 ± 0.18 ^{eC}	5.40 ± 0.01 ^{gC}	6.45 ± 0.02 ^{dC}	7.51 ± 0.10 ^{eB}
	Bastille	2.76 ± 0.06 ^{bcB}	3.47 ± 0.08 ^{aB}	4.53 ± 0.01 ^{bB}	6.46 ± 0.07 ^{dB}	8.38 ± 0.05 ^{ghA}

Dutchess	2.90 ± 0.07 ^{deC}	3.85 ± 0.04 ^{bB}	5.26 ± 0.01 ^{fC}	5.89 ± 0.07 ^{bC}	7.76 ± 0.01 ^{fB}
Faro	3.01 ± 0.06 ^{efC}	3.77 ± 0.02 ^{bB}	5.19 ± 0.03 ^{efC}	6.59 ± 0.08 ^{deB}	8.32 ± 0.11 ^{gB}
Jessie	3.30 ± 0.05 ^{gC}	4.79 ± 0.03 ^{eB}	4.86 ± 0.04 ^{cB}	5.72 ± 0.08 ^{aB}	5.80 ± 0.07 ^{aA}
Oro de Valle	2.45 ± 0.05 ^{aA}	4.42 ± 0.08 ^{dC}	5.84 ± 0.09 ^{hC}	7.82 ± 0.05 ^{hC}	8.55 ± 0.06 ^{hB}
Pasto	–	–	–	–	–
Puno	3.67 ± 0.07 ^{hC}	4.41 ± 0.06 ^{dC}	6.11 ± 0.06 ^{iC}	7.01 ± 0.04 ^{gB}	7.66 ± 0.11 ^{efD}
Rouge Marie	2.71 ± 0.03 ^{bA}	4.36 ± 0.03 ^{dA}	4.39 ± 0.07 ^{aA}	6.06 ± 0.02 ^{cA}	6.30 ± 0.05 ^{bcA}
Summer Red	2.87 ± 0.03 ^{cdC}	4.10 ± 0.02 ^{cC}	4.33 ± 0.10 ^{aC}	5.95 ± 0.03 ^{bcC}	6.38 ± 0.13 ^{cB}
Titicaca	3.71 ± 0.02 ^{hiC}	5.14 ± 0.01 ^{fC}	6.28 ± 0.02 ^{jC}	6.72 ± 0.06 ^{efB}	7.31 ± 0.06 ^{dB}
Vikinga	3.82 ± 0.02 ^{iC}	4.34 ± 0.03 ^{dB}	5.12 ± 0.04 ^{deC}	5.96 ± 0.07 ^{bcB}	6.11 ± 0.08 ^{bA}
Zwarte	3.08 ± 0.10 ^{fB}	4.92 ± 0.10 ^{eB}	5.01 ± 0.04 ^{dB}	6.86 ± 0.08 ^{fgB}	7.55 ± 0.07 ^{eB}

[1] Within years, average values followed by the same lowercase letter are not significantly different ($p > 0.05$). Capital letters compare the three years for the same variety, average values followed by the same letter are not significantly different ($p > 0.05$). [2] WAI: water absorption index.

Table S2. Pasting parameters of thirteen quinoa wholemeal flours obtained from seeds grown under North-West European field conditions in 2017, 2018 and 2019 ($n = 3$).

Year	Variety	Pasting Temperature (°C) [1]	First Peak Viscosity (mPa.s) [1]	Peak Temperature (°C)	Breakdown (mPa.s) [1]	Second Peak Viscosity (mPa.s) [1]	Final Viscosity (mPa.s) [1]	Total Setback (mPa.s) [1]
2017	Atlas	62.29 ± 0.59 ^{abcB}	2234 ± 32 ^{deB}	95.10 ± 0.01 ^{cdA}	318 ± 15 ^{eC}	2350 ± 30 ^{deC}	1852 ± 25 ^{dB}	−64 ± 4 ^{cA}
	Bastille	–	–	–	–	–	–	–
	Dutchess	64.14 ± 0.91 ^{cdB}	2465 ± 53 ^{fC}	95.06 ± 0.02 ^{eA}	176 ± 22 ^{cB}	2687 ± 31 ^{fB}	1977 ± 65 ^{eB}	−311 ± 31 ^{aA}
	Faro	64.10 ± 0.58 ^{cdA}	2081 ± 32 ^{cC}	95.20 ± 0.03 ^{abC}	577 ± 31 ^{gB}	2379 ± 23 ^{eB}	1992 ± 42 ^{eA}	488 ± 50 ^{eA}
	Jessie	63.60 ± 0.47 ^{bcdB}	1878 ± 33 ^{bC}	95.11 ± 0.00 ^{cdB}	124 ± 11 ^{bC}	2185 ± 37 ^{cC}	1404 ± 30 ^{bB}	−350 ± 13 ^{aA}
	Oro de Valle	61.36 ± 0.41 ^{abA}	2134 ± 13 ^{cB}	95.22 ± 0.03 ^{abB}	643 ± 12 ^{hC}	2287 ± 13 ^{dB}	2036 ± 16 ^{eA}	545 ± 18 ^{eA}
	Pasto	64.27 ± 0.58 ^{cdA}	2560 ± 11 ^{gB}	95.10 ± 0.01 ^{cdA}	348 ± 8 ^{eA}	3193 ± 31 ^{gB}	2364 ± 26 ^{gB}	152 ± 20 ^{dA}
	Puno	62.53 ± 0.65 ^{abcA}	2161 ± 14 ^{cdC}	94.58 ± 0.17 ^{aB}	635 ± 39 ^{hC}	2617 ± 30 ^{fA}	2180 ± 46 ^{fA}	654 ± 79 ^{fA}
	Rouge Marie	–	–	–	–	–	–	–
	Summer Red	65.10 ± 0.75 ^{dB}	1696 ± 38 ^{aC}	95.09 ± 0.01 ^{dA}	−78 ± 17 ^{aA}	2156 ± 44 ^{cC}	1492 ± 44 ^{bcC}	−282 ± 25 ^{aA}
	Titicaca	60.42 ± 0.76 ^{aA}	2272 ± 45 ^{eC}	95.21 ± 0.03 ^{abA}	519 ± 21 ^{fC}	2338 ± 32 ^{deB}	1860 ± 22 ^{dA}	108 ± 11 ^{dA}
	Vikinga	60.86 ± 0.41 ^{aB}	1732 ± 46 ^{aC}	95.14 ± 0.01 ^{bcA}	262 ± 17 ^{dC}	1787 ± 32 ^{aB}	1297 ± 32 ^{aA}	−173 ± 25 ^{bA}
	Zwarte	63.40 ± 0.92 ^{bcdA}	1728 ± 17 ^{aB}	95.11 ± 0.01 ^{cdA}	143 ± 5 ^{bcB}	1979 ± 20 ^{bB}	1542 ± 21 ^{cB}	−43 ± 10 ^{cA}
2018	Atlas	66.14 ± 0.26 ^{cdeC}	1824 ± 50 ^{dA}	95.16 ± 0.00 ^{bA}	276 ± 14 ^{fB}	1879 ± 43 ^{cA}	1566 ± 42 ^{bcA}	19 ± 7 ^{aB}
	Bastille	66.92 ± 0.87 ^{deB}	2492 ± 42 ^{gA}	95.18 ± 0.01 ^{bA}	384 ± 20 ^{gA}	3118 ± 54 ^{hB}	2817 ± 76 ^{gB}	708 ± 55 ^{eB}
	Dutchess	64.71 ± 1.06 ^{bcdB}	1729 ± 54 ^{cB}	95.13 ± 0.06 ^{bA}	66 ± 6 ^{dA}	2032 ± 39 ^{dA}	1956 ± 45 ^{dB}	293 ± 16 ^{cdB}
	Faro	67.11 ± 0.60 ^{eB}	2020 ± 27 ^{fB}	93.69 ± 0.19 ^{aA}	410 ± 9 ^{gA}	2685 ± 41 ^{gC}	2646 ± 40 ^{fB}	1037 ± 24 ^{fC}
	Jessie	64.06 ± 0.11 ^{bcB}	1030 ± 22 ^{aB}	95.13 ± 0.00 ^{bB}	−251 ± 13 ^{aA}	1627 ± 34 ^{bB}	1500 ± 34 ^{bC}	220 ± 15 ^{cB}
	Oro de Valle	62.98 ± 1.73 ^{abA}	2085 ± 29 ^{fB}	95.06 ± 0.26 ^{bB}	317 ± 15 ^{fB}	2565 ± 22 ^{fC}	2522 ± 23 ^{eB}	753 ± 26 ^{eB}
	Pasto	65.60 ± 1.40 ^{cdeA}	1915 ± 20 ^{eA}	95.14 ± 0.00 ^{bA}	378 ± 2 ^{gA}	2288 ± 26 ^{eA}	1914 ± 18 ^{dA}	378 ± 18 ^{dB}
	Puno	62.75 ± 0.34 ^{abAB}	1900 ± 57 ^{deB}	95.16 ± 0.04 ^{bC}	155 ± 29 ^{eB}	2734 ± 28 ^{gB}	2721 ± 32 ^{fB}	976 ± 48 ^{fB}
	Rouge Marie	–	–	–	–	–	–	–
	Summer Red	66.44 ± 0.14 ^{deB}	1092 ± 27 ^{aB}	95.09 ± 0.01 ^{bA}	−81 ± 14 ^{cA}	1432 ± 13 ^{aB}	1238 ± 12 ^{aB}	64 ± 14 ^{abB}
	Titicaca	60.65 ± 0.18 ^{aA}	1924 ± 22 ^{eB}	95.08 ± 0.01 ^{bA}	65 ± 15 ^{dB}	2579 ± 30 ^{fC}	2542 ± 23 ^{eC}	683 ± 48 ^{eC}
	Vikinga	62.86 ± 0.36 ^{abC}	1303 ± 14 ^{bB}	95.14 ± 0.00 ^{bA}	−187 ± 8 ^{bB}	1877 ± 26 ^{cC}	1624 ± 20 ^{cB}	133 ± 15 ^{bB}
	Zwarte	–	–	–	–	–	–	–
2019	Atlas	59.87 ± 1.39 ^{abcA}	1871 ± 27 ^{gA}	95.05 ± 0.02 ^{cdA}	45 ± 19 ^{fgA}	2273 ± 37 ^{fB}	2069 ± 45 ^{ghC}	242 ± 32 ^{bcC}
	Bastille	64.34 ± 0.23 ^{eA}	2453 ± 21 ^{iA}	95.16 ± 0.00 ^{dA}	513 ± 12 ^{iB}	2601 ± 16 ^{gA}	2119 ± 15 ^{hA}	178 ± 40 ^{bA}
	Dutchess	61.80 ± 1.14 ^{cdA}	1656 ± 31 ^{fA}	95.12 ± 0.03 ^{cdA}	94 ± 20 ^{gA}	1996 ± 30 ^{dA}	1843 ± 17 ^{eA}	281 ± 12 ^{cB}
	Faro	63.77 ± 0.90 ^{deA}	1959 ± 21 ^{hA}	94.19 ± 0.24 ^{abB}	555 ± 17 ^{iB}	2135 ± 17 ^{eA}	1965 ± 15 ^{fA}	560 ± 4 ^{dB}
	Jessie	59.31 ± 1.24 ^{abA}	823 ± 15 ^{aA}	94.62 ± 0.09 ^{bcA}	−53 ± 21 ^{dB}	1200 ± 7 ^{aA}	1142 ± 6 ^{bcA}	267 ± 15 ^{cB}

Oro de Valle	61.51 ± 1.28 ^{bcdA}	1677 ± 19 ^{fA}	94.24 ± 0.86 ^{abA}	239 ± 7 ^{hA}	2026 ± 7 ^{dA}	2020 ± 7 ^{fgA}	582 ± 17 ^{dA}
Pasto	–	–	–	–	–	–	–
Puno	64.31 ± 0.24 ^{eB}	1654 ± 25 ^{fA}	93.99 ± 0.25 ^{aA}	−364 ± 49 ^{aA}	3078 ± 26 ^{hC}	3047 ± 28 ^{iC}	1030 ± 58 ^{eB}
Rouge Marie	62.10 ± 0.68 ^{cdeA}	908 ± 13 ^{abA}	94.93 ± 0.24 ^{cdA}	−17 ± 12 ^{deA}	1206 ± 47 ^{aA}	1098 ± 18 ^{abA}	173 ± 9 ^{bA}
Summer Red	62.59 ± 1.15 ^{deA}	940 ± 15 ^{bA}	95.22 ± 0.02 ^{dA}	−41 ± 8 ^{deB}	1183 ± 2 ^{aA}	1027 ± 15 ^{aA}	46 ± 11 ^{aB}
Titicaca	61.44 ± 0.32 ^{bcdA}	1293 ± 23 ^{eA}	95.12 ± 0.00 ^{dA}	−202 ± 10 ^{bA}	2170 ± 4 ^{eA}	2096 ± 3 ^{ghB}	601 ± 20 ^{dB}
Vikinga	57.70 ± 0.75 ^{aA}	1151 ± 24 ^{dA}	95.13 ± 0.00 ^{dA}	−147 ± 7 ^{cA}	1720 ± 14 ^{cA}	1600 ± 15 ^{dB}	303 ± 41 ^{cC}
Zwarte	63.02 ± 1.22 ^{deA}	1054 ± 17 ^{cA}	95.11 ± 0.01 ^{cdA}	1 ± 2 ^{efA}	1361 ± 19 ^{bA}	1225 ± 16 ^{cA}	172 ± 20 ^{bB}

[1] Within years, average values followed by the same lowercase letter are not significantly different ($p > 0.05$). Capital letters compare the three years for the same variety, average values followed by the same letter are not significantly different ($p > 0.05$).

Table S3. Pearson correlation analysis between physicochemical properties [1].

	WAI 55 °C	WAI 65 °C	WAI 75 °C	WAI 85 °C	WAI 95 °C	PV1	HS	BD	PV2	FV	TSB
WAC	0.758 **	0.616 **	0.560 **	0.373 *	n.s. [2]	−0.419 *	n.s. [2]	−0.432 *	n.s. [2]	n.s. [2]	n.s. [2]
WAI 55 °C		0.675 **	0.627 **	0.364 *	n.s. [2]	−0.454 **	n.s. [2]	−0.463 **	n.s. [2]	n.s. [2]	n.s. [2]
WAI 65 °C			0.744 **	0.553 **	n.s. [2]	−0.457 **	−0.361 *	−0.371 *	n.s. [2]	n.s. [2]	n.s. [2]
WAI 75 °C				0.809 **	0.395 **	n.s. [2]	n.s. [2]	n.s. [2]	n.s. [2]	0.445 **	0.693 **
WAI 85 °C					0.607 **	n.s. [2]	n.s. [2]	n.s. [2]	n.s. [2]	0.447 **	0.771 **
WAI 95 °C						0.457 **	0.340 *	0.398 **	0.551 **	0.747 **	0.734 **
PV1							0.848 **	0.736 **	0.856 **	0.647 **	n.s. [2]
HS								n.s. [2]	0.895 **	0.706 **	n.s. [2]
BD									0.415 *	n.s. [2]	n.s. [2]
PV2										0.902 **	0.433 *
FV											0.748 **

* Correlation significant at $p < 0.05$ level. ** Correlation significant at $p < 0.01$. [1] WAC: water absorption capacity, WAI: water absorption index, PV1: first peak viscosity, HS: holding strength, BD: breakdown, PV2: second peak viscosity, FV: final viscosity, TSB: total setback [2] n.s.: not significant

Table S4. Results of ANOVA (factors, *p* value).

Variable[1]	Variety	Year	Variety × Year
WAC	$p < 0.001$	$p < 0.001$	$p < 0.001$
WAI 55 °C	$p < 0.001$	$p < 0.001$	$p < 0.001$
WAI 65 °C	$p < 0.001$	$p < 0.001$	$p < 0.001$
WAI 75 °C	$p < 0.001$	$p < 0.001$	$p < 0.001$
WAI 85 °C	$p < 0.001$	$p < 0.001$	$p < 0.001$
WAI 95 °C	$p < 0.001$	$p < 0.001$	$p < 0.001$
pasting temperature	$p < 0.001$	$p < 0.001$	$p < 0.001$
first peak viscosity	$p < 0.001$	$p < 0.001$	$p < 0.001$
peak temperature	$p < 0.001$	$p < 0.001$	$p < 0.001$
breakdown	$p < 0.001$	$p < 0.001$	$p < 0.001$
second peak viscosity	$p < 0.001$	$p < 0.001$	$p < 0.001$
final viscosity	$p < 0.001$	$p < 0.001$	$p < 0.001$
total setback	$p < 0.001$	$p < 0.001$	$p < 0.001$

[1] WAI: water absorption capacity; WAI: water absorption index
